# Monthly Summary

**Published:** 1898-12

**Authors:** 


### Monthly Summary.

COMMITTEES OF THE NATIONAL DENTAL ASSOCIATION.—Section I.—Prosthetic Dentistry, Metallurgy and Chemistry: I. N. Broomell, chairman; Wm. E. Walker, secretary.

Section II.—Dental Education, Literature and Nomenclature: S. H. Guilford, chairman; M. F. Finley, secretary.

Section III.—Operatlve Dentistry: J. Y. Crawford, chairman; Frank Holland, secretary.

Section IV.—Histology and Microscopy: T. L. James, chairman; L. L. Dunbar, secretary.

Section V.—Materia Medica and Therapeutics: J. S. Cassidy, chairman; A. W. Harlan, secretary.

Section VI.—Physiology and Etiology: J. D. Patterson, chairman; L. E. Custer, secretary.

Section VII.—Anatomy, Pathology and Surgery: W. C. Barrett, chairman; W. F. Lewis, secretary.

Section VIII.—Hygiene and Prophylactic Dentistry: J. Taft, chairman, H. B. McFadden, secretary.

Section IX.—Orthodontia: V. H. Jackson, chairman; C. L. Goddard, secretary.

Section X.—Clinics: H. J. McKellops, chairman; W. B. Culver, secretary.

GEO. H. CUSHING,
Recording Secretary National Dental Association.

---

HEMORRHAGE AFTER EXTRACTION.—Dr. Gledhill says to control undue hemorrhage after extraction apply to the alveolus, until full, pledgets of cotton thoroughly saturated with phenol sodique dipped in tannic acid. Hold finger on last piece one or two minutes to compress it.—*Dental Weekly.*

NATURAL LAW AND ITS PENALTIES.—We are about to resume the study of some phases of natural law as applied to the human organism and of those diseases, disintegrations, or dissolutions which are the penalty for its violation—a penalty inexorably demanded alike from the obliviousness of ignorance or the unheedfulness of knowledge; and demanded, too, not only from the guilty living, but from those who guiltlessly inherit them.

With tireless research the science of medicine has sought to make Nature's secret thought her own, and to impart to all men that knowledge by which they may at least be saved from ignorant violation of her mandates and its attendant penalties.

In that beneficent labor dentistry has borne her fitting share, and has dedicated to humanity results which need not fear comparison with those achieved by any other branch of medicine. But while we are thus assembled to learn yet more of the lessons which science has taught, we cannot as citizens be unmindful of the kindred but broader lesson which history is teaching—that in the moral as in the physical world, that for nations as for men, there can be no sin without its penalty.

Even now across tropic seas this cosmic law echoes from the cannon's mouth, as a once great but cruel and now decadent nation meets her Nemesis.

On the waves of those waters first ploughed by the prows of her adventurous ships, and on the shores of that queenly isle which she has reddened with the blood of misruled peoples and of murdered guests, her expiation is appointed. Thus have decreed the fateful Three who spin the thread of destiny.

And He who holds nations and destinies and seas and shores in the hollow of His hand has to our nation, which desired only peach, given " not peace but a sword," unheathable until retributive justice is satisfied.

May the duty thus assigned to our reluctant hands be fulfilled bravely, as becomes freemen; wisely, as becomes a sovereign people; mercifully, as becomes Humanity,—Items of Interest.

FILLING WITH GOLD OVER CEMENT.—When a man undertakes the task of criticising adversely the work or opinion of others, he should be very sure that he thoroughly understands his own position and that he so expresses himself that others may also comprehend his meaning. Let us see how Dr. Wedelstaedt measures up to this standard as a critic. The controvery on this point begins on page 647 of Items of Interest where he says:

"Men tell us it is easy to place cement into cavities and then pack amalgam upon and into it. I have made some experimental fillings of this kind in the cavity block. The cavities were not filled three-quarters full of soft cement, but about one-quarter. After the first piece of amalgam was crowded into the soft cement, the margins were most carefully cleaned, all excess amalgam and cement was removed and the packing of amalgam proceeded with, as has been suggested in this chapter. These fillings have been examined by twenty or twenty-five dentists who have, without exception, pronounced them perfect. And they are so, as far as one can judge from appearances. But examine them with the one-inch power in the microscope and you have their true condition revealed. And what is it? Margins of cement."

In response to this Dr. Clapp sent to me a tooth partly filled with gold overlying cement, and after examining the same I published the following opinion (page 789):

"I must report that in the tooth sent his margins are quite free of cement. Furthermore, it seems to me that there is no reason to apprehend trouble of this character if the dentist is careful not to use an excessive quantity of cement. The method is good if properly applied."

This language seems simple, explicit and readily understood, yet Dr. Wedelstaedt has not comprehended it since he appears to attribute to the words a wilder significance than they can possibly have. In connection with Dr. Clapp's specimen, all that I say is that "his margins are clean," whereas after examining the tooth with much more care than I, Dr. Wedelstaedt himself says (page 809), "The margins of this cavity are devoid of any cement."

Then Dr. Wedelstaedt goes on to inform us that the cement used by Dr. Clapp was in poor condition, porous granular and cracked; that Weston's cement is worthless anyway; that cements in general have little " crushing stress," etc., etc., *ad nauseam*, and is " greatly surprised," that I overlooked all this. There need be no surprise as I could not have overlooked that for which I made no search. All that I examined was the margins, and in that respect Dr. Wedelstaedt admits all that was sought to be proven, viz.: that by this method clean margins could be preserved, a position which he antagonized in the first criticism.

My closing statement that " the method is good if properly applied " has no reference whatever to the specimen of Dr. Clapp's work, but is based upon a clinic experience in my own practice covering many years; an experience of continued success which engenders a faith in the method in no wise shattered by the pseudo scientific investigations by which Dr. Wedelstaedt arrives at the conclusion that cements have little " crushing stress."

The method " properly applied " depends not upon the resistance which the material exerts under pressure, but solely upon its adhesive qualities as a cement. In short, the plastic is utilized to retain the substratum of gold in large cavities, so that the operator may readily attach the first pieces of gold, but after this is admitted, two points must be prominently observed. First, the cement may be relied upon for retaining the first layers of gold only, never for retaining the filling. (See methods of Filling Teeth, page 148). Second, whether much or little of the cement be used (depending upon the need of utilizing it as an insulator) that portion of the cavity which is to be occupied by the metallic filling should be so fashioned that the forces of mastication cannot be transmitted to the underlying mass of cement. Thus it becomes absolutely immaterial whether the cement has little or great " crushing stress," if only the method be "properly applied."—Items of Interest.

PAIN AND THE SURGEON.—Many surgeons study pain in a somatic more than they do in a psychic way. It is as essential to study the various phases of psychic as of bodily pain. Mental shocks in the weakened and suffering have debarred cure. It is not necessary to create a castastrope to shock those mentally weak, but we venture to assert that the rough indifference of the surgeon and the display of instruments have, combined, killed more than the mere operation. Trivialities carefully studied lead to perfection. Picture, if you will, a weak, delicate, suffering woman, whose brain dwells in morbid sensibility. She comes to a surgeon for operative procedure. Our lordly man of the knife has not time to indulge in the ordinary amenities of life and examines his patient in orderly, business fashion, without the faintest show of sympathy or feeling and tells in the shortest possible way what has to be done, and honestly says that he cannot tell what the result may be. He has not time to soothe, placate or encourage. The time comes for the operation. The patient approaches the table in painful and nervous apprehension; nothing is said to encourage her. Chloroform is given without a soothing suggestion. Here is a mind that a mental shock can and will influence violently, and this mind goes under the influence of chloroform in a state where a morbid suggestion may be the means of causing grave results. It is a poor surgeon who cannot soothe and quiet a pained mind as well as a pained body. The surgeon is more often an unconscious inflicting agent, in operative work than he thinks.

A surgeon unacquainted with psychic pain is only half educated, and in truth the surgery of to-day becomes more mechanical and less soulful. Narrow, technique surgery makes most of its successes through the thoughts of other brains than that of the operator. If genius were required to make surgeons, then indeed they would be very few and far between. Technique is mechanical, not intellectual. The surgeon should not only be skilled in technique, but should have knowledge of every element of his art. He should be a student of the mind as well as of the body, and success only comes in its best form when we interpret every condition and surrounding of the patient.—The Railway Surgeon.

NATIONAL SCHOOL OF DENTAL TECHNICS.—The next annual meeting of the National School of Dental Technics will meet at Cincinnati, Ohio, on the 28th and 29th of December, 1898, beginning promptly at 10 a. m. with the address of President G. V. Black. The partially-made-up programme is as follows .

"The Value of a Graded Course of Study and Uniformity Among Dental Schools," by G. V. I. Brown; Reports of Syllabi Committees; "Operative Technics," by T. E. Weeks; "Prosthetic Technics," by N. S. Hoff; "Symposium of Teaching Methods," by W. H. Whitslar, C. M. Wright and H. H. Burchard; "Steel Technics," by G. H. Wilson; "Teaching Cavity Preparation," by C. N. Johnson; Master of Exhibits, Grant Molyneaux.

Discussion on the papers will be opened by prominent teachers. It is hoped that all interested in the newer methods of teaching in dental schools will be present. A profitable time is promised. Exhibits of class work will be interesting. The profession is cordially invited to attend. Meeting held in the club rooms of the Grand Hotel.

D. M. CATTELL, Sec'y-Treas.

---

A GOOD CEMENT.—An excellent cement for mending anything may be made by mixing together litharge and glycerin to the consistency of thick cream or fresh putty. The cement is useful in mending stone jars or any coarse earthenware, stopping leaks in seems of tin pans or wash boilers, cracks and holes in iron kettles, etc. It may also be used to fasten on lamp tops, or tighten loose nuts, to secure loose bolts whose nuts are lost, to tighten loose joints of wood or iron, or in many other ways about the various kitchen utensils, the range, sink, and in the pantry fittings. In all cases the article mended should not be used till the cement has hardened, which will require from one day to a week, according to the quantity of cement used. This cement will resist the action of water, hot or cold, acids, and almost any degree of heat.—Dental Brief.

PRESERVATION OF ETHER.—The loss caused by the evaporation of ether from unsealed bottles during hot whether, and tin shops that are kept very warm in winter, can be prevented by the following ingenious plan: Fill the bottle nearly tull with ether, pour in sufficient glycerin to bring the surface of the ether nearly to the bottom of the stopper; then insert the latter carefully, and with a piece of twine of sufficient length suspend the bottle neck downward from a nail or other support, letting the cord pass over the stopper and take a turn around the body of the bottle from each side, so that it will hang perpendicularly and safely. The glycerin descends and fills the neck of the bottle, sealing the latter hermetically, and when any of the contents are required for use it is necessary only to reinvert the bottle, remove the stopper, and carefully wipe the neck before pouring. The glycerin of course drops to the bottom and allows the ether to be poured out almost to the last drop if care be taken. A similar plan is useful for bottles containing benzol.—National Druggist.

---

PLASTER IMPRESSIONS AND MODELS.—The writer says he adds a small quantity of vermillion to impression plaster to enable him to distinguish the impression from the model. For easy separation of the two, he coats the impression with a thin mixture of linseed oil and vaseline.—F. H. GOFFE.

[Excellent results are obtained by coating the impression with thin shellac varnish; after drying place in water while mixing the plaster; take from the water and shake off the surplus and pour the plaster.—ED. *Dental Weekly*.

---

A CHEAP METHOD OF CROWNING.—Loop a platinum wire round the root and take impression with loop in position, cast in plaster and remove impression material; fit a plain backed tooth to model, squeeze the pins to loop, invest and solder with silver solder. Clean and replace on model, then build up masticating portion of crown with amalgam, forcing the amalgam well down into the root, polish when hardened. Roots too badly decayed to crown by any other system can be saved by this method.—*Dr. Barnes*, *Leghorn*, *Italy*.

MERCURY, TO PURIFY.—Dr. Beacock says hydrochloric acid has no effect on mercury, sulphuric acid must be heated to affect it much; nitric acid acts on it lightly; by taking advantage of this, mercury can be purified easily from lead and many other base metals or impurities with which it is often mixed. Using one part acid to eight parts water, heated to 140 F., will not attack the mercury, and ls sufficiently strong to eat up the baser metals the mercury may contain, Another way to purify mercury is to shake it well in pulverized sugar, then filter through a paper cone by making pin holes in the bottom of it. The mercury will filter through, leaving the sugar in the paper.—*Dental Weekly.*

---

TREATING FOUL PULP-CANALS.—Dr. Jackson, of Michigan, says he finds the following dressing very valuable in treating foul pulp-canals with soreness at the end of root:

Menthol pip. crystal,
Choral hydrate, equal parts.

It is somewhat antiseptic, is soothing, agreeable to taste and smell.

He says also that chloral hydrate is a solvent for camphor, which makes a solution of value to dentists.—Dental Weekly.

---

REFLECTED LIGHT FOR THE OPERATING CHAIR.—Fasten a white shade on spring roller in the usual manner to the top of the window. To reflect the light down on the chair, draw the shade out horizontally by means of a cord passing through a pulley suspended from the ceiling or to the wall at the rear of the chair.—Dental Weekly.

---

TO SOLDER CAP ON GOLD CROWN.—To solder a cap on a gold tube intended for an artificial crown, lay the cap on about a tablespoonful of finely cut asbestos. put the tube in place on the cap, drop in the solder and a little powdered borax, then blow a yellow flame all around the tube till the solder flows, and there will be no danger of melting the plate. —J. G. Templeton.

---

FITTING PORCELAIN INLAYS.—The most important point to be observed in fitting a porcelain inlay is to mix the cement, used for holding it in position, as thin as possible; if it be mixed too thick, it loses its tenacity, prevents the tapering inlay being pressed fully home, and a bad fit is the result. Chlora-percha can in many cases be successfully used for fixing inlays in position; it is not affected by the fluids of the mouth.—Ash's Quarterly.

---

SANDPAPER YOUR JOINTS.—If you remove gum blocks from the flask, and rub the joints very lightly over fine sandpaper before replacing them, they can be packed much cleaner. Where there is any vestige of wax there will be unclean joints.—Dom. Journal.

---

CARIOUS DENTIN.—When through removal of carious dentin would expose the pulp, I leave a layer, covering it with a thin slice of gutta-percha dipped in chloroform sufficiently to dissolve the surface and make it adhesive.—Dr. Chas. Sill, in Dental Brief.

---

PACKING VULCANITE.—If you pack vulcanite warm and wet, it packs clean as well as easy.—Dominion Dental Journal.

